# Adult outcomes following neonatal oesophageal atresia: more reasons to be concerned?

**DOI:** 10.1038/s41390-026-04764-4

**Published:** 2026-01-14

**Authors:** Katherine B. Frayman, David G. Tingay

**Affiliations:** 1 Respiratory Diseases Group, Murdoch Children’s Research Institute, Parkville, VIC Australia; 2 Respiratory and Sleep Medicine, Royal Children’s Hospital, Melbourne, VIC Australia; 3 Department of Paediatrics, University of Melbourne, Melbourne, VIC Australia; 4 Neonatal Research, Murdoch Children’s Research Institute, Parkville, VIC Australia; 5 Department of Critical Care, University of Melbourne, Melbourne, VIC Australia

Oesophageal atresia (OA), with or without tracheoesophageal fistula, is a rare developmental anomaly of the foregut, affecting between 1 in 3500 to 1 in 4100 live births. Affected infants undergo surgical repair in the first days to weeks of life, and with advances in surgical and neonatal care, >90% survive into adulthood. There is, however, significant long-term multisystem morbidity, including respiratory, when compared to other neonatal developmental conditions. Specifically, OA is associated with congenital airway anomalies, classically tracheomalacia (nearly universal). Oesophageal dysmotility and swallowing difficulties are commonly associated with oropharyngeal dysphagia and primary and/or secondary aspiration.^1^ Airway clearance is  impaired, and there is increased morbidity, including hospitalisation, associated with childhood respiratory infections. The focus of long-term care has been related to oesophageal and tracheal calibre and function.

Adult survivors of OA describe chronic respiratory morbidity, including wheeze, productive cough and reduced exercise tolerance.^2^ Pulmonary function testing reveals both obstructive and restrictive deficits,^3^ however the pathophysiological contribution of each factor is unclear. The study of Aubert and colleagues helps address this knowledge gap.^4^ The authors describe the chest wall characteristics (motion and anatomical deformities) and lung volumes in 30 adults (median [IQR] 28.7 [23.0, 40.0] years; 17 female) who had a right thoracotomy repair of OA in the neonatal period, and compare these to 22 control adults without OA (31.3 [25.6, 36.9] years; 15 female). The type of OA was representative of the OA population, with 70% Type C, 17% Type A, 7% Type B and 3% Type D. To address their study aims they used real-time MRI (rt-MRI). Not surprisingly, they found that rib adhesions (40%) or fusion (50%) and scoliosis (43.3% of OA group) were more common in adults with OA, especially moderate to severe anatomical scoliosis. Right lung volumes were also less. The findings were similar to a previous cohort of children studied with the same methods, except scoliosis was more common in adults (43% versus 14%).^5^

Rt-MRI is a relatively new modality that allows dynamic, real-time imaging of anatomical structures in motion without the need for contrast medium or heavy sedation. The authors have considerable experience with this technique. In their current study they used rt-MRI for three purposes: 1) assessing chest wall motion during normal breathing, 2) calculating lung volumes in the right and left lung and 3) defining anatomy in the thorax (including vertebra). The rates of chest wall abnormalities were much higher than other follow up studies,^6^ highlighting a potential for rt-MRI to understand pathophysiology that is not possible with standard imaging and measurement techniques. It is important to consider that structure is not the same as function, and whilst adults with OA had a high rate of moderate to severe anatomical scoliosis the majority were not symptomatic. Pairing rt-MRI with current assessment tools will be needed to determine if existing approaches are inadequate.

In all participants the original OA repair was by a right thoracotomy.^4^ This likely explains the isolated right rib abnormalities. These findings are consistent with an earlier study by the same authors, which applied rt-MRI to 50 children with repaired OA (mean age 9.8 ± 4.0 years), although scoliosis was less prevalent in children (overall prevalence 14%).^5^ Interestingly, in the earlier study, there were no differences in thoracic volume or motility in a small group who had undergone minimally invasive surgery (*n* = 8) compared with a larger cohort of survivors managed with open thoracotomy repair (*n* = 42).^5^ A recent systematic review exploring musculoskeletal outcomes following thoracoscopic versus open repair of OA identified four retrospective studies, including a total of 283 patients (thoracoscopic repair *n* = 96, open repair *n* = 187), including those of the authors’ previous study, and reported a reduction in scoliosis and rib anomalies, but not chest wall deformities or scapula alata, associated with thoracoscopic repair.^7^ Clearly neonatal surgery is needed to ensure survival and respiratory and gastrointestinal independence in OA. But these results raise the question as to how surgery in a state of significant developmental immaturity may alter growth and function trajectories, and then how do clinicians balance often conflicting priorities and risks. Thoracoscopic repair of OA is an accepted alternative but remains less common than an open thoracotomy.^8^ It is logical that less manipulation of the developing ribs would reduce abnormalities but until adequately sized long-term follow-up cohorts are studied caution must be applied. Such studies also need to balance the neonatal outcomes of different surgical approaches.

At all levels of the chest, participants demonstrated reduced cross-sectional area (volume) of the right hemithorax relative to their own left hemithorax, and to that of controls. Importantly, chest wall motion was not uniformly impaired in the right lung. Only the right upper and middle chest wall, and not the lower, had reduced motion suggesting localised functional impairments. OA is not usually associated with fetal hypoplasia and the impact of the right-sided rib anomalies on postnatal lung growth and mechanics could explain the reduced right lung volumes. Vital capacity of the right lung, assessed using rt-MRI volume coverage technique, was reduced relative to the participants’ left lung and to controls.

Impairment in respiratory function is frequently described in children, adolescents and adults with repaired OA. Cohort studies utilising conventional lung function techniques, including spirometry and plethysmography, report restrictive, obstructive and mixed defects, with prevalence ranging from 11 to 67%, 7 to 54% and 4 to 13%, respectively.^3^ Risk factors for their development, and potential targets for prevention and/or management, are less clear. A multicentre retrospective national cohort study of 503 patients with type C (termed III in study) OA born 2008–2013, including 216 with interpretable spirometry, found only 63.4% had normal lung function, with restrictive, obstructive and mixed defects in 26.9%, 5.1% and 4.6% of participants, respectively.^9^ Patient-associated factors, including birth weight, congenital heart defects, tracheomalacia and neonatal gastro-oesophageal reflux, were associated with restrictive spirometry; surgical approach and musculoskeletal abnormalities, as reported on chest radiograph, were not.^9^ In the study of Aubert and colleagues the smaller right lung volumes were statistically significant but not dramatically different in absolute terms at end-inspiration. Ideally, pairing rt-MRI with functional tools (such as spirometry), clinical history and existing measure of skeletal assessments will aid in understanding the clinical implications of rt-MRI lung measures.

There is always a risk of selection bias with follow up studies. Without significant resources, populations tend to include those more likely to have symptomatic features. In this study, the true representativeness of the OA population is unclear in terms of the underlying population number, rates of lost to follow-up or mortality (and why) and the underlying co-morbidities and operative details (no access to medical files). This may explain the relatively low rates of comorbidities such as VACTERL.

To minimise morbidity, and maximise long-term health outcomes, multidisciplinary care and a cohesive approach to monitoring and surveillance is required. This includes regular and co-ordinated reviews by respiratory medicine, otolaryngology, speech pathology, gastroenterology and paediatric surgery, with input from physiotherapy, dietetics and social work as required. Routine surveillance oesophagoscopy with biopsies is recommended during infancy, childhood and adolescence;^10^ flexible and rigid bronchoscopy and formal evaluation of swallow are recommended in infancy, and then as required.^11^ Regular pulmonary function testing is indicated, with cross-sectional imaging (CT chest) for individuals at risk of bronchiectasis, postulating that, as has been achieved in other chronic respiratory conditions of childhood, early diagnosis, understanding of the pathophysiological contributors, and targeted interventions, will ultimately improve long-term outcomes.

The work of Aubert and colleagues reinforces the need for integrated and structured follow-up of OA throughout life. It highlights how hard it is to achieve this, requiring commitment across multiple silos of healthcare that are rarely interlinked (neonatal critical care, paediatrics and then adult systems). It also requires communication between the different allied health care and medical and surgical sub-specialities mentioned earlier. The importance of respiratory and oesophageal dysmotility (aerodigestive) monitoring in childhood is well understood.^10,11^ The current manuscript by Aubert and colleagues highlights the additional importance of routine musculoskeletal surveillance beyond just scoliosis. Hopefully, further studies will integrate existing respiratory and digestive assessment tools with rt-MRI to develop individual morbidity profiles and identify preventable or modifiable factors (including surgical approach). Fundamentally, parents need to be aware of the long-term risks and supported as they navigate their children into adulthood and beyond.
